# Cerebrospinal fluid neurofilament light chain protein levels in subtypes of frontotemporal dementia

**DOI:** 10.1186/1471-2377-13-54

**Published:** 2013-05-29

**Authors:** Maria Landqvist Waldö, Alexander Frizell Santillo, Ulla Passant, Henrik Zetterberg, Lars Rosengren, Christer Nilsson, Elisabet Englund

**Affiliations:** 1 Section of Geriatric Psychiatry, Department of Clinical Sciences, Lund University Hospital, Klinikgatan 22, Lund SE-221 85, Sweden; 2 Section of Pathology, Department of Clinical Sciences, Lund University, Lund, Sweden; 3 Institute of Neuroscience and Physiology, Department of Psychiatry and Neurochemistry, The Sahlgrenska Academy at the University of Gothenburg, Mölndal, Sweden; 4 UCL Institute of Neurology, Queen Square, London WC1N 3BG, United Kingdom; 5 Institute of Neuroscience and Physiology, Department of Clinical Neuroscience and Rehabilitation, The Sahlgrenska Academy at the University of Gothenburg, Gothenburg, Sweden

**Keywords:** Semantic dementia, Neuropathology, Clinical diagnosis

## Abstract

**Background:**

Frontotemporal dementia (FTD) is recognised as a clinically and morphologically heterogeneous group of interrelated neurodegenerative conditions. One of the subtypes within this disease spectrum is the behavioural variant FTD (bvFTD). This is known to be a varied disorder with a mixture of tau-positive and tau-negative underlying pathologies. The other subtypes include semantic dementia (SD), which generally exhibits tau-negative pathology, and progressive non-fluent aphasia (PNFA), which is usually tau-positive. As the clinical presentation of these subtypes may overlap, a specific diagnosis can be difficult to attain and today no specific biomarker can predict the underlying pathology. Neurofilament light chain protein (NFL), a cytoskeletal constituent of intermediate filaments, is thought to reflect neuronal and axonal death when appearing in the cerebrospinal fluid (CSF). NFL has been shown to be elevated in CSF in patients with FTD compared with AD and controls. Our hypothesis was that the levels of NFL also differ between the subtypes of FTD and may indicate the underlying pathological subtype.

**Methods:**

We retrospectively analysed data from previous CSF analyses in 34 FTD cases (23 bvFTD, seven SD, four PNFA), 20 AD cases, and 26 healthy controls. A separate group of 10 neuropathologically verified and subtyped FTD cases (seven tau-negative, three tau-positive) were also analysed.

**Result:**

NFL levels were significantly higher in FTD compared with both AD (p<0.001) and controls (p<0.001). The NFL levels of SD and bvFTD were significantly higher (p<0.001) compared with AD. The biomarker profiles of PNFA and AD were similar. In the neuropathologically verified FTD cases, NFL was higher in the tau-negative than in the tau-positive cases (exact p=0.017).

**Conclusions:**

The marked NFL elevation in some but not all FTD cases is likely to reflect the different underlying pathologies. The highest NFL values found in the SD group as well as in the neuropathologically verified tau-negative cases may be of subtype diagnostic value, if corroborated in larger patient cohorts. In bvFTD, a mixture of tau-positive and tau-negative underlying pathologies could possibly explain the intermediate NFL values.

## Background

Frontotemporal dementia (FTD) is a heterogeneous group of neurodegenerative conditions affecting the frontal and temporal lobes and, to a varying degree, the subcortical grey matter. In the 1998 consensus document, FTD was divided into a number of clinical subtypes: behavioural variant (bvFTD) and progressive aphasias, semantic dementia (SD) and progressive non-fluent aphasia (PNFA) [1]. There is a close association, both clinically and neuropathologically, with corticobasal degeneration (CBD) and progressive supranuclear palsy (PSP); thus these diagnoses are now generally considered part of the FTD spectrum [2].

There is considerable overlap between the different subtypes of FTD, both clinically and neuropathologically. A major challenge in diagnosing FTD is the difficulty in predicting a neuropathological subtype based on clinical characteristics. With reference to protein pathology, cases of FTD have previously been recognised as tau-positive or tau-negative. Among the tau-positive pathologies, cases of Pick’s disease and FTD with tau-positive inclusions have been found. The majority of tau-negative cases have been ubiquitin-positive. In 2006, TDP-43 was identified in the ubiquitin-positive inclusions in the majority of these cases [3] and, recently, FUS-positive pathology was identified in most of the remaining cases [4,5]. The remaining small group exhibits a spectrum of variable morphological factors [6,7]. There is not always a clear congruence between the clinical and neuropathological subtypes of FTD. While bvFTD can reflect any of the neuropathological subtypes, SD almost exclusively shows TDP-43 pathology and PNFA most often shows tau-positive inclusions [8]. FTD with concomitant motor neuron disease (FTD-MND) most often exhibits TDP-43 pathology.

In AD, the analysis of cerebrospinal fluid (CSF) biomarkers (with a typical pattern of elevated tau, phosphorylated tau [phospho-tau] and decreased beta-amyloid 42 [Aβ42]) is an acknowledged and promising tool in the diagnosis of Alzheimer’s disease [9]. However, there are no reliable CSF biomarkers available for FTD in general or for the different FTD subtypes. A previous study demonstrated that CSF levels of TDP-43 were slightly increased in amyotrophic lateral sclerosis (ALS) compared with controls [10], but its role in the clinical diagnosis of FTD is yet to be determined.

Neurofilament proteins, when prevalent in the CSF, are considered to reflect neuronal degeneration. These intermediate filaments form a major cytoskeletal constituent of neurons and they comprise the neurofilament heavy (NFH), intermediate (NFM) and light (NFL) chain proteins, the latter being a non-phosphorylated form.

NFL levels are slightly augmented in healthy aging individuals and have been correlated with increasing age [11]. In acute cerebral infarction, very high NFL levels can be seen. The levels are higher in VaD than in AD [12]. A recent study [13] showed that NFL levels were significantly higher in atypical parkinsonian disorders (CBD and multiple system atrophy) compared with Parkinson’s disease; whereas NFL levels in Lewy body dementia were intermediate. The suggested explanation for these differences was that patients with atypical parkinsonian disorders experienced a greater loss of neurons over a shorter period of time. NFL levels are also increased in individuals with white matter disease (WMD) [14]. WMD is common in patients with dementia (AD, among other diseases), but can also be seen in healthy older adults [15]. WMD is indicated by hyperintensities visualised during life as punctuate or confluent changes in the periventricular regions or deep white matter on computerized tomography or magnetic resonance imaging scans [15,16].

Some studies have shown that CSF levels of NFL are higher in FTD compared with both early-onset AD and controls [17,18]. In ALS it has also been shown that NFL is often considerably increased compared with controls [19,20], and elevated levels of NFL correlate with shorter survival [19].

The aim of this study was to investigate whether existing CSF biochemical markers, in particular NFL, can assist in the differential diagnostics of frontotemporal dementias.

Can biomarkers help us differentiate between clinical and/or neuropathological subtypes of frontotemporal dementias? Furthermore, is the severity of the disease post mortem related to in vivo NFL levels?

## Methods

### Patients/study population

This study focused on two populations: 1. a clinical cohort of 34 FTD cases, 20 AD cases and 26 healthy controls and 2. a separate cohort of 10 post mortem verified FTD cases.

We re-evaluated the clinical files of all patients who had been diagnosed with FTD, SD or progressive non-fluent aphasia (PNFA) at the Memory Clinic, Lund University Hospital and who had undergone a lumbar puncture between 2002 and May 2010. A prerequisite for inclusion was that patients’ neurofilament light chain protein (NFL) levels had been previously analysed. The method used to analyse NFL changed in June 2010; hence only patients with CSF samples taken before this time were included. Patients with a clinical diagnosis of PSP or CBD were not included.

### Clinical revision and subtyping

The clinical diagnoses of 44 eligible FTD patients were revised and divided into subgroups according to the 1998 consensus criteria on FTD [1] by two independent observers (one senior consultant [CN]) and one senior neuropsychologist. Ten patients were excluded on the basis of an uncertain diagnosis, as they either did not meet the clinical criteria or they presented other types of brain lesions, i.e. strategic infarctions on neuroimaging, or other lesions that could better explain their symptoms. In three cases, the conclusion of the two observers differed and a consensus diagnosis was reached after revision. The revised diagnoses were determined by observers blinded to the CSF examination results. The initial case selection, however, was established from the existing medical records based on clinical diagnoses at the time of investigation. Therefore at this initial stage the observers were not formally blinded to the CSF examination results. These results, however, were not pivotal for the diagnosis at this stage, but served to demonstrate that none of the SD or bvFTD individuals had a CSF profile typical of AD (high phospho-tau, low beta-amyloid).

Of the 34 patients included in the study, 23 patients were diagnosed with bvFTD. Among these, two patients did not fully meet all five core criteria of any of the existing subgroups of FTD, but the suspicion of a disorder within the FTD spectrum was strong and was further supported by neuroimaging. The clinical characteristics in these two cases were closest to the behavioural variant. One of the two patients met the criteria of probable FTD according to the recently suggested clinical criteria of bvFTD [21]. Seven cases were diagnosed as SD and four cases as PNFA based on existing clinical criteria [1].

A group of 20 age-matched AD patients were selected as controls among the patients who had undergone a diagnostic LP including NFL measurement between 2006–2008 and who met the NINCDS-ADRDA criteria [22] for probable AD.

All patients, both within the FTD and AD groups, had given their written consent to save CSF for research purposes (biobank no. SC57; BD15; registry no. 136). Additionally, the majority of the FTD patients were part of ongoing longitudinal studies for which the Regional Ethical Review Board at Lund University had given their approval (dno. 617/2008, 16/2011, 137/2012). No new CSF analyses were performed specifically for this study; instead, the results from the clinical investigations were used.

NFL data from a group of 26 age- and sex-matched healthy controls were retrieved from research material previously published by Zetterberg et al. 2007 [19].

A neuropathologically studied group of 10 FTD patients, who during life had undergone a lumbar puncture with measurement of NFL levels, was identified. The cases were all morphologically diagnosed and typed at the Department of Pathology at Lund University Hospital between 2001 and 2009.

Demographic data for both patients (age at onset, age at LP, gender) and the control group (age at LP, gender) was noted (Table 1).

**Table 1:** Demographic data for study population

**Group**		**N**	**Male/Female**	**Median duration of dementia at time of LP (range)**	**Median age at LP (range)**	**Median duration from LP to death (range)**
FTD		34	15/19	3 (1–9)	70 (38–85)	-
	bvFTD	23	12/11	3 (1–9)	72 (38–85)	-
	SD	7	2/5	2 (1–4)	64 (53–78)	-
	PNFA	4	1/3	3 (1–5)	69 (66–77)	-
AD		20	7/13	2 (1–7)	72 (49–84)	-
Healthy controls		26	12/14	NA	70 (56–83)	-
NP verified FTD		10	5/5	3 (0.4-12)	63 (32–78)	2 (0.1-8)
	Tau-pos	3	1/2	5 (4–7)	69 (50–78)	3 (2–4)
	Tau-neg	7	4/3	3 (0.4-12)	60 (32–67)	2 (0.1-8)

### CSF analysis

CSF collection and analysis had been performed in a clinical setting prior to this study, according to the standardised procedure at the Memory Clinic in Lund. In two cases, the lumbar puncture was carried out at nearby Memory Clinics with similar routine procedures. CSF was obtained by lumbar puncture and centrifuged at 2000 *g* at +4°C for 10 min within 2 h of sampling. It was then aliquoted, and stored in polypropylene tubes at −80°C without being thawed and refrozen prior to biochemical analysis*.* Basic CSF analyses of cells, protein, sp-Alb and the Alb ratio were performed at the Department of Clinical Chemistry, Lund University Hospital. The albumin ratio was calculated as CSF albumin (mg/L)/serum albumin (g/L) and was used as a measure of blood–brain barrier function [23]. Analyses of tau, p-tau, Aβ42 and NFL were performed at the Clinical Neurochemistry Laboratory at the Sahlgrenska University Hospital in Mölndal, Sweden, according to previously described ELISA procedures [11,24-26]. The detection limit of the NFL assay was 250 ng/L.

### Neuropathology

Prior to this study, all 10 cases were examined and diagnosed by the same neuropathologist (EE) according to the diagnostic procedures at the Department of Pathology, Lund. These procedures included whole brain assessment with entire bi-hemispheric coronal sections covering all major regions for conventional staining, including silver stains and additional small area sections for immunohistochemical assessments of protein pathology. The neuropathology procedure in Lund has been described previously in detail [27]. For the present study, the 10 cases were revised for confirmation. The overall severity of degeneration was noted as mild, moderate or severe, judged from macroscopically assessed regional atrophy and from microscopically assessed grading of cortical degeneration [28,29]. This grading was made on hematoxylin-eosin stained sections from all cortical regions. Mild degeneration connotes a superficial shrinkage and reduction of neurons mainly in cortical layer II, accompanied by a glial reaction in layers I to III, with or without a mild laminar microvacuolation. Moderate degeneration reveals a deeper engagement of cortical layers, with some reduction of layer V neurons. Severe degeneration represents a pancortical degeneration with advanced neuronal loss and a variable but more intense gliosis accompanied by marked limbic, especially anterior cingulate and hippocampal, degeneration [28,29]. Furthermore, complementary sectioning and staining for the detection of pathological TAR DNA-binding protein 43 (pTDP-43, Cosmo Bio Ltd., Tokyo, Japan) and the protein fused in sarcoma (FUS, Sigma-Aldrich LLS, St. Louis, MO, USA) were performed. Sections with tau and ubiquitin staining were previously available.

### Statistics

The NFL values of the clinical group cases were analysed for each clinical subtype. The NFL values of the neuropathologically diagnosed FTD cases were analysed for different protein pathologies and demographic features. We used the detection limit (250 ng/L) as the lowest possible value of NFL in the statistical analyses.

All analyses were carried out in SPSS 19.0. Between-group differences in CSF biomarkers were analysed using the non-parametric Kruskal-Wallis test followed by the Mann–Whitney U test (exact p-value and corrected for ties). Each group was analysed separately. A p-value of <0.05 was considered to be statistically significant. When presenting results for the smallest groups, the exact p-value is given. The Spearman rank test was used to check for potential correlations. Because of the small size of the sample populations, the demographic data as well as the levels of biomarkers are presented as medians and range (min – max).

## Results

### Demographic data

Demographic data for the study population (both patient groups and the clinical and neuropathological material) are summarised in Table 1. At the time of LP, the median age of the AD group was slightly greater than that of the FTD group, whereas the duration of disease was somewhat shorter (non-significant differences).

### CSF biomarkers in the clinical patient group

**Figure 1:**
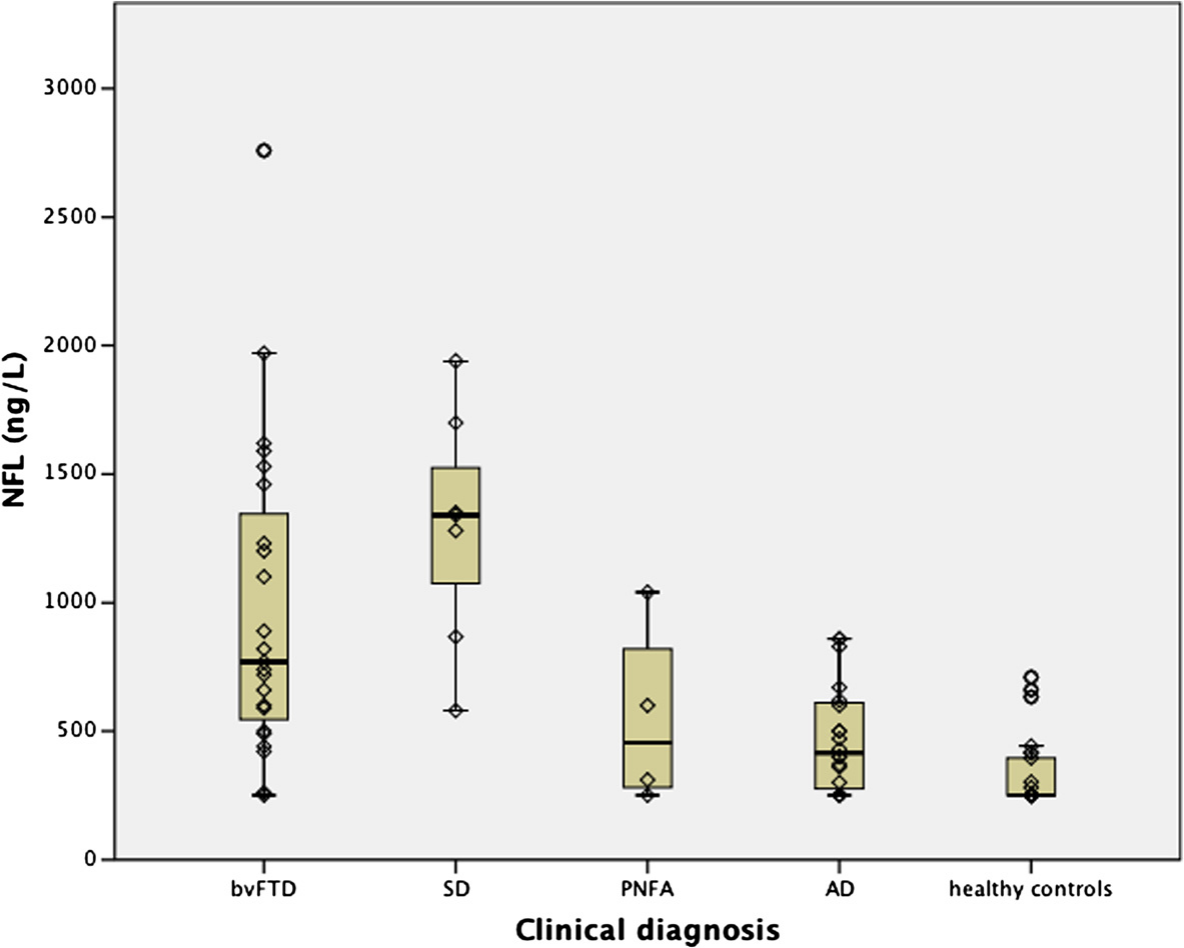
**Boxplot with individual values of NFL in each clinical subtype.** The number of subjects in each subtype: bvFTD n=23, SD n=7, PNFA n=4, AD n=20, healthy controls n=26. The detection limit of NFL was 250 ng/l.

The median NFL level for the entire FTD group was significantly higher than for both the AD group (p<0.001) and the healthy controls (p<0.001). NFL levels in both bvFTD and SD, but not PNFA, were significantly higher (p<0.001) than in AD (Figure 1 and Table 2). Within the FTD group, the NFL median level was highest in SD, intermediate in bvFTD and lowest in PNFA, however, the difference was not statistically significant (asymptotic p-value 0.068). The following number of NFL samples were at or below the detection limit in each subgroup: bvFTD n=1, SD n=0, PNFA n=1, AD n=5, healthy controls n=17.

**Table 2:** The clinically investigated group - diagnosis and levels of CSF biomarkers, median (range)

	**FTD (n=34)**	**bvFTD (n=23)**	**SD (n=7)**	**PNFA (n=4)**	**AD (n=20)**	**Controls(n=26)**	**Significance**
NFL (ng/L)	844 (250–2760)	770 (250–2760)	1340 (580–1940)	455 (250–1040)	415 (250–860)	250 (250–710)	FTD> AD p<0.001 SD>AD p<0.001 FTDbv> AD p<0.001
Aβ42 (ng/L)	555 (140–1130)	570 (260–1130)	595(360–1110)	215 (140–380)	285 (140–460)	NA	FTD>AD p<0.001
Tau (ng/L)	355 (150–820)	280 (160–750)	385(180–770)	610 (150–820)	630 (230–2290)	NA	AD>FTD p<0.001
p-tau (ng/L)	49 (23–115)	45(29–82)	55 (23–92)	88 (45–115)	87 (42–237)	NA	AD<FTD p<0.001
Albumin ratio	0.00755 (0.0025-0.0346)	0.00765 (0.0043-0.0346)	0.00655 (0.0051-0.0134)	0.0058 (0.0025-0.0081)	0.00665 (0.0033-0.0099)	NA	Non-significant

*NA* Not analysed.

Median levels of tau, Aβ42 and phospho-tau (p-tau) are presented in Table 2. Aβ42 was significantly higher in the FTD group as a whole compared with the AD group, while tau and p-tau levels were significantly lower (p<0.001). The median levels of all three proteins were similar for bvFTD and SD, while the median levels for the PNFA group were similar to the AD group values. These differences were not statistically significant, probably because of the small group sizes. There was no correlation between NFL levels and the albumin ratio.

### CSF biomarkers in the neuropathological patient group

**Figure 2:**
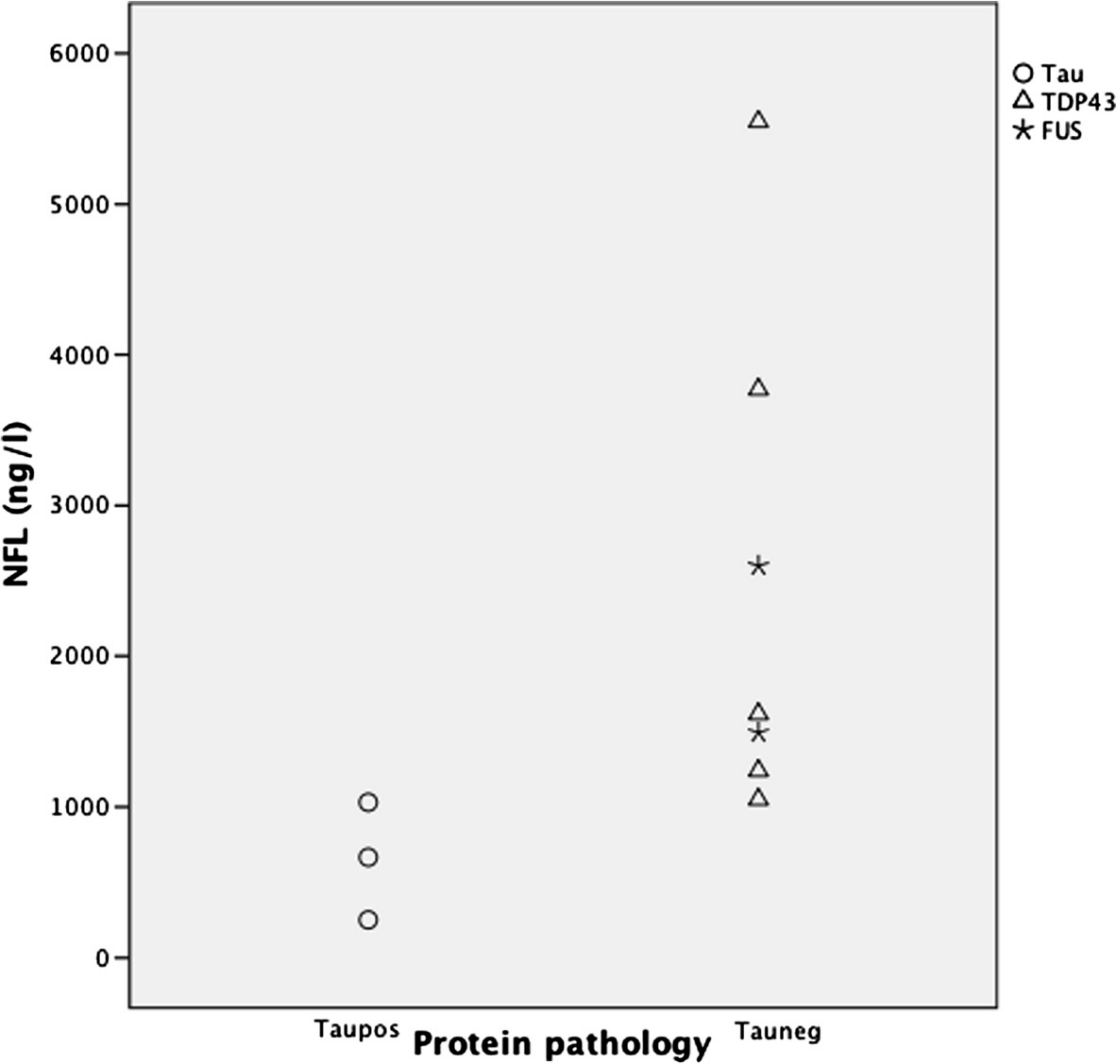
**Scatter of NFL values in each neuropathological subtype.** The number of subjects in each subtype: tau-positive n=3, tau-negative n=7. The detection limit of NFL was 250 ng/l.

In the patients with neuropathologically verified FTD, although limited in the number of cases, NFL values were significantly higher in the tau-negative cases (median 1620; range 1050–5546 ng/L) compared with the tau-positive cases (median 665; range 250–1030 ng/L) (p=0.017), as shown in Figure 2. There was no correlation between the assessed severity of cortical degeneration and levels of NFL.

In these cases, there was no correlation between the duration of dementia at the time of LP and the levels of any of the pathological proteins (Table 3). Furthermore, there was no association between CSF biomarker levels and neuropathological severity of degeneration, nor between these markers and brain weight.

**Table 3:** The neuropathologically investigated group - diagnoses and individual levels of CSF markers

**Case**	**Aβ42 (ng/L)**	**Tau (ng/L)**	**P-tau (ng/L)**	**NFL (ng/L)**	**Clinical diagnosis**	**Neuropathological diagnosis**	**Degeneration severity**	**Dementia duration at LP (years)**	**Age at death (years)**	**Brain weight (g)**
1	726	819	NA	5546	FTD	FTD-TDP43	moderate	2	54	1020
2	570	410	35	3770	FTD-MND	FTD-TDP43	mild	0.4	62	1600
3	420	620	NA	2600	bvFTD	FTD-FUS	severe	3	37	NK
4	500	450	54	1620	bvFTD	FTD-TDP43	severe	NK	67	1340
5	662	494	NA	1494	bvFTD	FTD-FUS	severe	3	34	NK
6	411	263	NA	1241	FTD(PPA)	FTD-TDP43	severe	12	76	975
7	370	330	29	1050	FTD(PPA)	FTD-TDP43	severe	5	69	760
8	780	490	68	1030	bvFTD	FTD-tau	mild	4	81	1125
9	369	554	95	665	FTD(PPA)	FTD-tau	severe	7	73	1010
10	639	528	NA	<250	AD	FTD-tau	mild	NK	53	1335

*PPA* Primary progressive aphasia.

*NA* Not analysed.

*NK* Not known.

## Discussion

To our knowledge, this study is the largest to date performed on CSF neurofilament light chain protein concentrations in clinically diagnosed FTD, and also the first study on NFL levels in neuropathologically verified FTD with pathological subtyping. Previous studies have shown that NFL levels are higher in FTD compared with both AD and controls [18]. Our results replicate those findings and extend previous data by reporting NFL values in different clinical and neuropathological subtypes of FTD.

The results of the present study need to be interpreted with caution considering the significant differences in group size between the diagnostic subtypes. The clinical diagnosis of patients was established through a rigorous process to minimise the risk of misdiagnosis. However, as the clinical diagnosis does not always reflect the underlying pathology, it is possible that some of the patients in the clinically diagnosed group did not have tau-positive or -negative frontotemporal lobar degeneration. A strength of this study is that we compared a group of patients with a clinical diagnosis of FTD with patients who had a neuropathologically verified diagnosis.

Our clinical data demonstrated that NFL levels were significantly higher in SD and bvFTD, but not PNFA, compared with both AD and controls. Within the FTD group the NFL levels were highest in SD; however, the differences between subgroups did not reach statistical significance. This is possibly because of the small number of cases in the SD and PNFA groups*.*

NFL values at the detection level (250 ng/L) were mainly found in the AD and control groups. The possibility that these values could have been even lower is not thought to have influenced the comparison between groups as we used non-parametric tests.

The similarities between CSF biomarkers in the small group of PNFA cases (n=4) and the AD group (n=20) suggest that there may be individuals with underlying Alzheimer pathology in the PNFA group, albeit with a clinical presentation of prominent non-fluent aphasia.

In the group with neuropathologically verified FTD, there were significant differences in NFL levels between subtypes; the tau-negative cases had higher NFL levels than the tau-positive cases. This fits with our finding in the clinical data that the SD group had the highest level of NFL, as this is the clinical presentation most often associated with TDP-43 pathology [30]. The clinical presentation of bvFTD can be associated with either a tau-positive or tau-negative neuropathology. This is in keeping with the intermediately increased levels of NFL in patients with bvFTD in this study compared with the PNFA and AD groups; the former of which are most often reported as tau-positive.

Neurofilament protein is a biomarker for neuronal death and axonal loss. It has previously been shown that rapidly progressive neurodegenerative disorders such as PSP and multiple system atrophy have higher mean levels of NFL than the more slowly progressive Parkinson’s disease [31]. From our clinical experience, moderate to severe regional atrophy on neuroimaging at the time of CSF sampling is often reflected in elevated NFL levels. In our neuropathological cases, however, there was no correlation between NFL levels at the time of lumbar puncture and the degree of degeneration and estimated overall severity at the time of death. In addition, blood–brain barrier dysfunction, measured by the CSF/serum albumin ratio, did not seem to contribute to the differences in NFL between subtypes. To establish whether high NFL levels in CSF reflect aggressive or rapid neuronal breakdown, longitudinal studies of clinical progression, neuroimaging and CSF sampling will need to be performed.

Two out of the 10 neuropathologically verified FTD cases showed high levels of NFL exceeding 3000 ng/L. In a previous study, a small fraction of patients within a larger set of clinically diagnosed patients with FTD had particularly high NFL levels [32]. Similarly, in a meta-analysis of CSF NFL levels in dementia, a small number of FTD patients in each study group had particularly high levels of CSF NFL and NFH [17]. As these authors suggested, it is likely that there is an important subgroup within the FTD spectrum with higher CSF NFL levels.

FTD-MND is exclusively associated with tau-negative pathology. There is a close relationship between FTD-MND and ALS, and several studies have shown high NFL levels in ALS, especially in the most rapidly progressing cases [19,20].

## Conclusions

NFL and other CSF biomarkers may be useful for the discrimination between different forms of FTD disorders, especially the primary progressive aphasias. Furthermore, NFL levels may indicate the underlying pathology, with a high NFL level corresponding to a tau-negative pathology, most often of the TDP-43 type. Further studies based on a larger population are needed to confirm our findings.

## Abbreviations

FTD: Frontotemporal dementia; bvFTD: Behavioural variant FTD; SD: Semantic dementia; PNFA: Progressive non-fluent aphasia; NFL: Neurofilament light chain protein; CSF: Cerebrospinal fluid; CBD: Corticobasal degeneration; PSP: Progressive supranuclear palsy; NFH: Neurofilament heavy chain protein; NFM: Neurofilament intermediate chain protein; TDP-43: TAR DNA-binding protein 43; FUS: Fused in sarcoma; ALS: Amyotrophic lateral sclerosis.

## Competing interests

The authors declared that they have no competing interests.

## Authors’ contributions

MLW participated in the design of the study, collected the data, performed the statistical analyses and drafted the manuscript. AFS participated in the design of the study and contributed to the collection of the data. UP participated in the design of the study and the clinical revision of diagnoses. HZ carried out the CSF analyses and together with LR contributed the control material. CN participated in the design of the study and revised the clinical diagnoses. EE participated in the design of the study, accounted for all neuropathological assessments and made major contributions to the manuscript draft. All authors read, contributed to and approved the final manuscript.

## Pre-publication history

The pre-publication history for this paper can be accessed here:

http://www.biomedcentral.com/1471-2377/13/54/prepub
